# Serum and CSF adiponectin, leptin, and interleukin 6 levels as adipocytokines in Egyptian children with febrile seizures: a cross-sectional study

**DOI:** 10.1186/s13052-016-0250-y

**Published:** 2016-04-12

**Authors:** Seham F. Azab, Mohamed A. Abdalhady, Mohamed A. A. Almalky, Ezzat K. Amin, Dina T. Sarhan, Eman M. Elhindawy, Mayy A. N. Allah, Ahmed A. Elhewala, Mohamed M. A. Salam, Mustafa I. A. Hashem, Attia A. Soliman, Nagwa E. Akeel, Sawsan H. Abdellatif, Nahla A. Elsamad, Anwar A. Rass, Manal S. Arafat

**Affiliations:** Faculty of Medicine, Zagazig University, 18 Omar Bin Elkhattab St, Al Qawmia, Zagazig City, AlSharqia Governorate Egypt; M.D. Clinical Pathology, Mansoura Student Hospital, Mansoura, Egypt

**Keywords:** Febrile seizure, adipocytokines, adiponectin, leptin, interleukin-6

## Abstract

**Background:**

A febrile seizure (*FS*) is the most common convulsive disorder in children. Activation of cytokine network is involved in *FS* pathogenesis. Adiponectin, leptin and IL-6 are the major adipocytokines secreted by fat cells. To date, only a few studies concerned the association of adipocytokines with febrile seizures. In this study, we tried to investigate serum and *CSF* levels of adiponectin, leptin, and interleukin-6 (*IL-6*); as adipocytokines, for the first time in Egyptian children with febrile seizures.

**Methods:**

This was a prospective cross-sectional study included one hundred patients with febrile seizure, and matched with age, gender, 100 children with febrile illness without seizures (febrile control, *FC*) and 100 healthy control group (*HC*). Serum and cerebrospinal fluid (*CSF*) levels of adiponectin, leptin, and (*IL-6*) were measured by enzyme-linked immunosorbent assay (*ELISA*) method.

**Results:**

Serum adiponectin was significantly higher in children with *FS* (16.8 ± 3.7 ug/ml) and the *FC* group (18.3 ± 4.3 ug/ml) compared to the *HC* group (9.5 ± 2.2 ug/ml); *P* < 0.05, respectively. Serum leptin was significantly lower in children with *FS* (0.9 ± 0.3 ng/ml) compared to both the *FC* group (4.7 ± 1.2 ng/ml) and the *HC* group (1.8 ± 0.4 ng/ml); *P* < 0.01, respectively. Children with *FS* had significantly higher serum IL-6 levels (43.7 ± 11.7 ng/ml) than the *FC* group (21.9 ± 4.5 ng/ml) and the *HC* group (6.5 ± 1.8 ng/ml); *P* < 0.01, respectively. Patients with simple febrile seizures *(SFS*) had serum and *CSF* adiponectin levels similar to those with complex febrile seizures (CFS); (*P* > 0.05). Serum and *CSF* leptin levels were significantly lower in patients with *CFS* compared to the *SFS* group (*P* < 0.05). Serum and *CSF* IL-6 levels were significantly higher in patients with *CFS* compared to the *SFS* group (*P* < 0.01). On multivariate logistic regression analysis, the high serum IL-6 levels was the most significant risk factor associated with febrile seizures among studied children (OR: 6.2; 95 % CI: 3.58 –10.57; *P* = 0.0001).

**Conclusion:**

Our data brought a novel observation that some adipocytokines like leptin and IL-6 could be, at least in part, an aetiopathogenetic factor in the manifestation of febrile seizures in susceptible Egyptian children. Moreover, we observed a significant association between high *CSF* IL-6 levels and susceptibility to complex febrile seizures as did the low *CSF* leptin levels.

## Background

Febrile seizures comprise common convulsive disorders in children between 6 months and 6 years of age, accounting for 30 % of all seizures in children [1]. Children with a simple febrile seizure have potential for recurrence and 2–7 % of children may develop epilepsy by adolescence [2]. Complex interaction between immune-inflammatory process, cytokines activation, and genetic factors is involved in febrile seizures pathogenesis [3]. The adipocytes are known to have an active endocrine function; adiponectin, leptin, interleukin-6 (IL − 6), tumor necrosis factor (*TNF − α*), vaspin, ometin and visfatin are produced in adipose tissue. These hormonally active peptides have common properties with cytokines, and therefore referred to as adipocytokines [4]. Adiponectin is a double-acting cytokine, which not only has anti-inflammatory effects, but it has also proinflammatory effects [5]. Adiponectin is protective against ischemic brain injury by modulating inflammatory pathway and endothelial functions and acts centrally to control peripheral metabolism [6]. Interestingly, PPARγ agonists, which are known to increase adiponectin expression, protect against seizure-related pathology [7]. Leptin affects energy homeostasis by decreasing food intake and by acting on lipogenesis and fatty acid oxidation [8]. Leptin has roles similar to proinflammatory cytokines [5]. Experimental work suggested that leptin might be an endogenous modulator of neuronal excitability [9]. Leptin has been proposed for clinical use as an anticonvulsant in addition to its potential metabolic effects in humans [10].

Pro-inflammatory and anti-inflammatory cytokines play an important role in regulating the febrile response during infection. Among these cytokines, interleukin-6 is the key acute-phase cytokine [11]. The association of IL-6 gene polymorphism and susceptibility to febrile seizures is still controversial. Increased levels of IL-6 have been reported in the plasma and cerebrospinal fluid of febrile seizure patients suggesting that IL-6 is activated during the acute stage of a febrile seizure [12, 13].

To date only a few studies concerned the association of adipocytokines with febrile seizures. In this study, we tried to investigate serum and *CSF* levels of adiponectin, leptin, and (IL-6); as adipocytokines, for the first time in Egyptian children with febrile seizures.

## Methods

This was a prospective cross-sectional study performed in Zagazig University Children Hospital, and outpatient clinics in the same hospital from May 2013 to October 2015.

One hundred children; who had febrile seizures as diagnosed in the Department of Pediatrics in the same hospital, were enrolled in this study. The age of the patients ranged from 6 months to 6 years (mean, 31 months). Diagnosis of febrile seizures followed the criteria established in the 1989 International Classification of Epileptic Syndromes [14]. The electroencephalogram (*EEG*) was normal for all patients or showed mild nonspecific abnormalities.

### Exclusion criteria

Patients with febrile seizures beginning at the age of 6 years or later, afebrile seizures, evidence of intracranial infection, epileptiform *EEG* traits or metabolic imbalance. Patients with diseases known to affect adipocytokines such as diabetes, genetic syndromes, and obese patients, were also excluded.

The mean age at the onset of febrile seizures was 18 months (range, 6–43months). Within the group with complex febrile seizures, 21 patients had experienced two or more seizures, five patients had experienced focal seizures, and nine patients had experienced prolonged seizures lasting longer than 20 min. Thirty-one patients had a family history of febrile seizures, and 4 patients had a family history of epilepsy.

Two hundred healthy children, of comparable age and gender; without a history of febrile or afebrile seizures, were enrolled as control groups. We subdivided our controls into two groups.

**FC group** (*n* = 100): children hospitalized at our pediatric department with fever due to infection except for central nervous system infection;

**HC group** (*n* = 100): who attended pediatric outpatient clinics for preoperative evaluation for elective surgery.

All patients and controls included were subjected to proper history taking, thorough clinical and detailed neurological examination. Laboratory investigations were done for all studied children and included: complete blood count (*CBC*) including blood indices, *ESR* and C-reactive protein (*CRP*), serum electrolytes (Na, K, Ca levels), blood glucose level, Urine culture and sensitivity tests, Liver function and kidney function tests.

Venous blood samples were obtained from patients within 30 min of the time of seizure, centrifuged and stored at _20 _C till the time of use. Control samples were collected and similarly analyzed. *CSF* samples were obtained from all *FS* patients based on the clinical judgment of the attending pediatrician. The *CSF* samples had normal cell count <5 cells/μl and normal protein levels (0.2–0.5 g/l).

### Serum and CSF adiponectin and leptin measurement

Serum and CSF adiponectin was measured by ELISA (AviBion, human adiponectin, Acrp30, Vantaa, Finland) that has assay range: 0.2 mg/L → 60 mg/L. Serum and CSF Leptin was detected with commercially available test kits were used to measure leptin (enzyme-linked immunosorbent assay [ELISA] method, DRG International, Mountainside, NJ, USA), that has assay range of 0.25 – 120 ng/mL.

### Serum and CSF interleukin 6 (IL6) measurements

The concentrations of IL6 in serum and CSF were estimated using a double antibody sandwich ELISA (kit provided by Biosource EuropeS.A., Belgium) according to the manufacturer’s instructions by using standard curve.

#### Statistical analysis

*SPSS* (version 22.0. Armonk, NY, USA: IBM Corp. 2013) was used for data analysis. The data are expressed as the mean ± *SD* or median (min-max) where appropriate. Test selection was based on evaluating the variables for normal distribution using the *Shapiro-Wilk test. Chi-square* test, *Student t test* and *ANOVA* test were used. Multiple comparison analysis by the least significant difference (*LSD*) was used. This test detects statistical difference between two means when *ANOVA* test refers to significances. Binomial logistic regression analysis was used to define the association between febrile seizures (as the dependent variable), estimated plasma adipocytokines levels and clinical parameters (as the independent variables). *P* value < 0.05 is considered to be statistically significant.

### Ethics

Informed parental consent was obtained to be eligible for enrollment into the study. The study was done according to the rules of the Local Ethics Committee of Faculty of Medicine, Zagazig University, Egypt. The study was approved by our local ethics committee of University of Zagazig, Egypt, reference number ZU_IRB #2597/–17–5–2013.

## Results

Our study included 100 patients with *FS* (65 patients with Simple *FS* and 35 patients with Complex *FS*, their age ranged from 6 to 72 months (mean 31 months), 51 males and 49 females) and 200 children as *FC* and *HC* groups whose clinical and laboratory characteristics are listed in Table 1. The control groups were age, gender and *BMI* matched to children with *FS*. Compared to the *FC* group, children with *FS* showed no significant differences in terms of body temperature on admission, type of infection (bacterial/viral), duration of fever before blood sample (<24 h/>24 h), white blood cell (*WBC*) count and C-reactive protein (*CRP*); all *P* > 0.05; (Table 1).

**Table 1:** Baseline clinical and laboratory characteristics of *FS* patients and the control groups

	FS group	FC group	HC group	P
	(*n* = 100)	(*n* = 100)	(*n* = 100)	
Age (months)	31 ± 7.5	33 ± 6.9	29 ± 8.3	>0.05
Gender (Male/female)^d^	51/49	50/50	48/52	>0.05
BMI (kg/m^2^)	15.9 ± 1.2^a^	16.3 ± 1.6^a^	15.7 ± 1.8^a^	>0.05
BT (C) on admission	38.9 ± 1.2	38.5 ± 1.3	_	>0.05
Type of infection ^d^ (bacterial/viral)	63/37	59/41	_	>0.05
Duration of fever ^d^ Before blood sample(<24 h/>24 h)	71/29	75/25	_	>0.05
*Type of Febrile seizure : Simple*/*Complex*	65/35	-	-	
WBC (× 10^9^/L)	13.5 ± 4.3^a^	14.1 ± 5.6^a^	8.7 ± 2.6^b^	<0.01
CRP (mg/dl)	38.4 ± 9.6^a^	41.0 ± 11.5^a^	3.8 ± 1.2^b^	<0.01
Serum Adiponectin (ug/ml)	16.8 ± 3.7^a^	18.3 ± 4.3^a^	9.5 ± 2.2^b^	<0.05
Serum Leptin (ng/ml)	0.9 ± 0.3^a^	4.7 ± 1.2^b^	1.8 ± 0.4^c^	<0.01
Serum IL-6 (ng/ml)	43.7 ± 11.7^a^	21.9 ± 4.5^b^	6.5 ± 1.8^c^	<0.01

Abbreviations: *FS* febrile seizures, *FC* febrile control, *HC* healthy control, *BMI* body mass index, *BT* body temperature, *WBC* white blood cell, *CRP* C-reactive protein, *IL-6* Interleukin 6

Values are mean ± standard deviations or median (minimum- maximum)

The *P* value is for *ANOVA*. ^a^, ^b^ and ^c^ on means refer to significant difference between means when *ANOVA* test refers to significances by multiple comparison analysis (aa, bb, cc = non-significant, ab, ac, bc = significant). ^d^Chi-square test

**Fig. 1:**
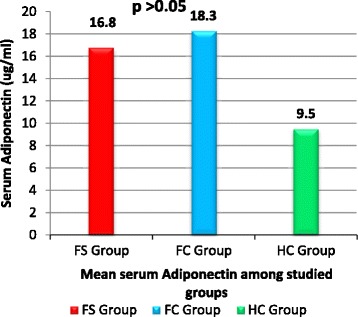
Serum adiponectin level among studied groups

**Fig. 2:**
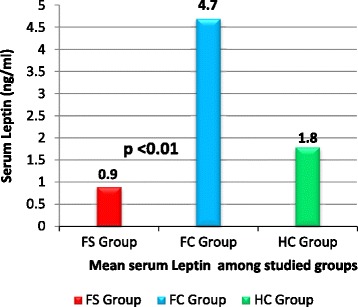
Serum leptin level among studied groups

Serum adiponectin was significantly higher in children with *FS* (16.8 ± 3.7 ug/ml) and the *FC* group (18.3 ± 4.3 ug/ml) compared to the *HC* group (9.5 ± 2.2 ug/ml); *P* < 0.05, respectively. However, no significant difference was observed between *FS* and *FC* groups as regards serum adiponectin; (*P* > 0.05), (Fig. 1). Serum leptin was significantly lower in children with *FS* (0.9 ± 0.3 ng/ml) compared to both the *FC* group (4.7 ± 1.2 ng/ml) and the *HC* group (1.8 ± 0.4 ng/ml); *P* < 0.01, respectively. On the other hand, serum leptin was significantly higher in the *FC* group compared to the *HC* group; (*P* < 0.01), (Fig. 2).

**Fig. 3:**
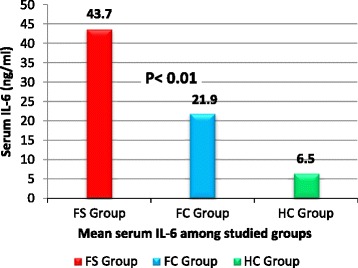
Serum IL-6 level among studied groups

Children with *FS* had significantly higher serum IL-6 levels (43.7 ± 11.7 ng/ml) than the *FC* group (21.9 ± 4.5 ng/ml) and the *HC* group (6.5 ± 1.8 ng/ml); *P* < 0.01, respectively; (Fig. 3).

Patients with simple febrile seizures had serum and *CSF* adiponectin levels similar to those with complex febrile seizures (*P* > 0.05); Table 2. Of note, serum and *CSF* leptin levels were significantly lower in patients with *CFS* compared to the *SFS* group (0.6 ± 0.07 vs 3.7 ± 0.8 ng/ml for serum leptin and 0.24 ± 0.08 vs 1.48 ± 0.3 ng/ml for *CSF* leptin, respectively; *P* < 0.05) Table 2. On the other hand, serum and *CSF* IL-6 levels were significantly higher in patients with *CFS* compared to the *SFS* group (55.8 ± 13.7vs 31.9 ± 7.6 ng/ml for serum IL-6 and 39.06 ± 5.7 vs 22.3 ± 6.8 ng/ml for *CSF* IL-6, respectively; *P* < 0.01) Table 2. Meanwhile, no significant differences were observed between *FS* patients’ subgroups as regards age, gender, BMI, body temperature on admission, duration of fever, WBC count or CRP (all *P* > 0.05; Table 2).

**Table 2:** Comparison between patients with simple febrile Seizure (SFS) and those with complex febrile seizures (CFS)

	SFS Group (*n* = 65)	CFS Group (*n* = 35)	*P*
Age (months)	30 ± 6.5	33 ± 7.2	>0.05
Gender (Male/female)^a^	34/31	17/18	>0.05
BMI (kg/m^2^)	15.6 ± 3.2	16.5 ± 3.1	>0.05
BT (C) on admission	38.7 ± 1.2	39.3 ± 1	>0.05
Duration of fever Before blood sample (<24 h/>24 h)	47/18	24/11	>0.05
WBC (× 10^9^/L)	14.7 ± 6.1	13.5 ± 3.4	>0.05
CRP (mg/dl)	40.2 ± 6.9	36.7 ± 5.3	>0.05
Serum Adiponectin (ug/ml)	3.1 ± 1.1	2.9 ± 0.7	>0.05
Serum Leptin (ng/ml)	3.7 ± 0	0.6 ± 0.07	<0.05
Serum IL6 (ng/ml)	31.9 ± 7.6	55.8 ± 13.7	<0.01
CSF Adiponectin (ug/ml)	1.8 ± 1.2	1.6 ± 0.9	>0.05
CSF Leptin (ng/ml)	1.48 ± 0.3	0.24 ± 0.08	<0.05
CSF IL6 (ng/ml)	22.3 ± 6.8	39.06 ± 5.7	<0.01

Abbreviations: *SFS* Simple febrile seizures, *CFS* Complex febrile seizures, *BMI* body mass index, *BT* body temperature, *WBC* white blood cell, *CRP* C-reactive protein, *IL-6* Interleukin 6, *CSF* cerebrospinal fluid

Values are mean ± standard deviations

*P* < 0.05 significant, (Student *t* test) ^a^Chi-square test

In the univariate logistic regression analysis high serum adiponectin, low serum leptin, and high serum IL-6 levels were significantly associated with the risk of febrile seizures among studied *FS* patients, but age, gender, body temperature on admission, type of infection, duration of fever, *WBC* count and *CRP* were not. When multivariate logistic regression analysis was performed, the high serum IL-6 levels was the most significant risk factor associated with febrile seizures among studied children (OR: 6.2; 95 % CI: 3.58 –10.57; *P* = 0.0001), but low serum leptin levels were also significantly associated with febrile seizures (OR: 2.2; 95 % CI: 1.09 –4.78; *P* = 0.047); Table 3.

**Table 3:** Multiple logistic regression analysis of serum adipocytokines levels as risk factors for febrile seizures among studied subjects

Risk Factor	Coefficient	*P* Value	Odds Ratio	*95 % CI* ^a^
Serum Adiponectin (ug/ml)	0.031	0.174	1.031	0.987- 1.078
Serum Leptin (ng/ml)	0.778 -	0.047	2.2	1.09 –4.78
Serum IL6 (ng/ml)	1.8171	0.0001	6.2	3.58 –10.57

^a^CI indicates confidence interval

## Discussion

Although febrile seizure is the most common form of seizures in children [15], its exact pathophysiology remains not fully understood. Several recent studies highlighted the role of cytokine network activation in febrile seizures etiopathogenesis [12, 13]. Recent experimental work suggested that peripheral endocrine and metabolic factors are capable of modulating seizure threshold and seizure-related pathology by acting on *CNS* neurons to trigger intracellular signaling pathways or modulating neuronal activity [7, 16]. This idea has raised a great deal of our interest in the role of the adipocytokines in the pathogenesis of febrile seizures. Adiponectin, leptin and IL-6 are the major adipocytokines secreted by fat cells [17].

Adiponectin is specifically expressed in human adipocytes. Adiponectin has multiple functions not only in the peripheral tissues, but also in the central nervous system. Adiponectin exerts its biological effects via its two receptors, and AdipoRs receptors, AdipoR1 and AdipoR2, are widely expressed in the central nervous system [18]. Central adiponectin has potent electrophysiological effects; raising the possibility that adiponectin directly modifies seizure activity and brain pathology [19]. In our study, we observed that serum adiponectin was significantly higher in children with *FS* and the *FC* group compared to the *HC* group. However, we couldn’t find any difference between *FS* and *FC* groups or between *SFS* and *CFS* patients in terms of serum adiponectin. Furthermore, patients with simple febrile seizures had *CSF* adiponectin levels similar to those with complex febrile seizures.

Similar to our results, a recent study by Güven et al. [20] who evaluated serum adiponectin levels in Turkish children with febrile seizures. This research suggested that elevated levels of serum adiponectin was an acute phase reactants in *FS* and *FC* groups that did not contribute to the development of *FS.*

Experimental work confirmed that adiponectin enhances tolerance against brain ischemia through anti-inflammatory and anti-oxidative mechanisms. Lee et al. [21] reported that adiponectin deficiency resulted in an increase in body fat, impaired glucose tolerance and increased lipids, and these changes were associated with increased seizure severity and hippocampal pathology. Previous studies suggested the neuroprotective action of adiponectin via an endothelial nitric oxide synthase-dependent mechanism [22]; meanwhile Chen et al. [6] confirmed the anti-inflammatory action of adiponectin against cerebral ischemia-reperfusion injury. However, little is known about the cerebroprotective action of adiponectin as well as its molecular mechanisms.

In the present study, children with *FS* had significantly lower serum leptin levels in comparison to both the *FC* group and the *HC* group; by contrast, febrile control children had significantly elevated serum leptin levels, compared to children with *FS* and healthy controls. Interestingly, serum leptin levels were significantly lower in patients with *CFS* compared to those with *SFS*. Our multivariate regression analysis model indicated that low serum leptin level was an independent risk factor associated with febrile seizures among studied children. In an attempt to explain our results concerning the anticonvulsant action of leptin, we studied the *CSF* level of leptin in our patients, which was significantly lower in patients with *CFS* compared to those with *SFS*. This finding has never been reported in children with *FS*. Our results were different from those of Khoshdel et al. [23] who reported no important change in serum leptin levels between children with simple febrile seizures and febrile children without seizures.

Leptin, a 16 kDa neurohormone predominantly synthesized and released into blood by adipocytes and serves as a signal for the brain of the body’s energy store [8]. Leptin controls food intake through its receptors in the hypothalamus by inhibiting the release of NPY, so leptin is a key hormone in the regulation of body weight and nutrition [8]. Recent experimental work confirmed leptin’s anticonvulsant action in seizure models. Xu et al. [10] reported that intranasal administration of leptin produced elevated brain and serum leptin levels and delayed the onset of pentylenetetrazole-induced generalized convulsive seizures. Leptin was found to inhibit ionotropic α-amino-3-hydroxy-5-methyl-4-isoxazole proprionic acid (AMPA) glutamate receptor–mediated synaptic transmission in hippocampal brain slices. This suppression of glutamate transmission by leptin can diminish the likelihood of seizure generation and propagation [10]. These findings, together with our results, support the hypothesis that the high serum leptin levels observed in *FC* group could be protective against febrile seizures, whereas lower serum leptin levels were associated with *FS* in susceptible children. The future may hold a greater promise for therapeutic success in using metabolic hormones and their signaling modalities to combat seizures disorders rather than obesity.

IL-6 is a pleiotropic proinflammatory cytokine with a wide range of biological activities in immune regulation, hematopoiesis, inflammation, and neoplasia and interleukin-6 demonstrates a strong correlation with fever [24]. A dual role of IL-6 in seizures has been demonstrated in *FS* experimental models [25–27]. An earlier study by Biber et al. [25] confirmed that stimulation of astrocytes and brain slices of cortex with IL-6 induced adenosine A1 receptor mRNA which is a powerful endogenous anti-convulsive substance. Fakuda et al. [26] reported that interleukin-6 plays an anti-convulsive role in experimental hyperthermia-induced seizures which might suggest similar properties of this cytokine in children with febrile seizures. On the other hand, intranasal administration of IL-6 exacerbated the severity of seizures induced by pentylenetetrazole on models of FS, supporting a pro-convulsant effect [27].

Our data revealed that children with *FS* had significantly higher serum IL-6 levels compared to both the *FC* group and the *HC* group. In addition, serum and *CSF* IL-6 levels were significantly higher in patients with *CFS* compared to the *SFS* group.

Our multivariate logistic regression model indicated that the high serum IL-6 level was the most significant risk factor associated with febrile seizures among studied children.

These results are concordant with those of Virta et al. [13] who found that plasma interleukin-6 levels and interleukin-1 receptor antagonist (IL-1 RA) were significantly higher in 55 patients with febrile seizures, compared with 20 age-matched, febrile control subjects. The authors recorded that elevated levels of IL-6, IL-1 RA and IL-10 were also found in the *CSF* of patients with *FS*, but *CSF* IL-6 levels were detectable in all studied patients with *FS*. Straussberg et al. reported an increase in the production of IL-1b, IL-6, and IL-10 cytokines by lipopolysaccharide-stimulated mononuclear cells from individuals of 13 children with history of febrile seizures and 11 controls, but the secretion of IL-6 and IL-10 in response to lipopolysaccharide was higher in those with a previous history of convulsions [28]. Hu et al. performed a study on 9 children with febrile seizures and 21 patients with severe acute encephalitis. In multivariate analysis, plasma IL-6 was significantly increased in patients with FS compared to those with severe acute encephalitis, suggesting that IL-6 is activated during the acute stage of a febrile seizure [12].

Our results confirm and extend these findings by demonstrating that elevated serum and *CSF* IL-6 levels in *FS* patients could be, at least in part, an aetiopathogenetic factor in the manifestation of febrile seizures in susceptible children. Lehtimäki et al. [29] explained that increased brain proinflammatory cytokines decrease the threshold for individual seizures; supporting the suggestion that neuroinflammation may contribute to epileptogenesis in the developing brain [29, 30]. However, final proof of a causal role of elevated serum and *CSF* IL-6 levels in febrile seizures pathogenesis is still lacking.

To the best of our knowledge, we demonstrated for the first time the association between serum and *CSF* levels of adiponectin, leptin, and (IL-6); as adipocytokines, and febrile seizures in Egyptian children. However, the small sample size was one of our limitations in this study; we suggest that multicenter approaches may be necessary to attain larger sample size. Interictal adipocytokines levels were not measured in our patients with febrile seizures which was another limitation in our study.

## Conclusion

Our data brought a novel observation that some adipocytokines like leptin and IL-6 could be, at least in part, an aetiopathogenetic factor in the manifestation of febrile seizures in susceptible Egyptian children. Moreover, we observed a significant association between high *CSF* IL-6 levels and susceptibility to complex febrile seizures as did the low *CSF* leptin levels.

Future more extended studies on febrile seizures investigating the serum and *CSF* levels of those and other adipocytokines; like *TNF − α*, vaspin, ometin and visfatin, will provide an additional understanding of the possible role of adipose tissue in the susceptibility to febrile seizure and progression to epilepsy.

## Abbreviations

CFS: Complex febrile seizure
CI: Confidence interval
CRP: C-reactive protein
CSF: Cerebrospinal fluid
EEG: Electroencephalography
ELISA: Enzyme-linked immunosorbent assay
FC: Febrile control
FS: Febrile seizure
IL-1: Interleukin-1
IL-1Ra: Interleukin-1 receptor antagonist
IL-6: Interleukin-6
OR: Odds ratio
PPARγ: Peroxisome proliferator-activated receptor γ
SFS: Simple febrile seizure
TNF − α: Tumor necrosis factor-α
WBC: White blood cell count
